# Association of prosocial personality traits with symptoms of depression, anxiety and stress in psychiatric nurses; Single-centre cross-sectional study in Croatia

**DOI:** 10.1192/j.eurpsy.2024.1730

**Published:** 2024-08-27

**Authors:** K. Bosak, I. Topolić Šestan, P. Folnegović Grošić, Ž. Bajić, I. Filipčić, V. Grošić

**Affiliations:** ^1^Psychiatric Clinic Sveti Ivan; ^2^Department of Psychiatry, University Hospital Centre Zagreb; ^3^Research Unit “Dr. Mirko Grmek”, Psychiatric Clinic Sveti Ivan, Zagreb, Croatia

## Abstract

**Introduction:**

Symptoms of depression, anxiety and stress are more common in the population of nurses working in psychiatric hospitals than in many other segments of the health care system. These three elements of psychological distress (depression, anxiety, stress) may reduce the nurse’s ability to establish quality therapeutic relationships with patients, which are very important in the treatment of mental disorders. Some studies suggest that prosocial personality traits may have a protective role. Other research suggests that high levels of empathy, for example, may increase secondary traumatisation and lead to more pronounced symptoms of distress.

**Objectives:**

The main objective of the study was to examine the association of prosocial personality traits with symptoms of depression, anxiety and stress in nurses employed in a psychiatric clinic. The hypothesis was that more pronounced prosocial personality traits are associated with a lower expression of symptoms of depression, anxiety and stress.

**Methods:**

The target population were nurses employed in a psychiatric hospital working directly with patients. No sample was selected, but the whole available population was invited to participate. The independent variable was prosocial personality traits measured by the Prosocial Personality Battery (PSB). The outcome was symptoms of distress (depression, anxiety and stress) measured using the Depression, Anxiety, Stress Scale-21 (DASS-21). The hypothesis was tested using three linear regression analyses.

**Results:**

Total of 63 MST were included with a median (interquartile range) age of 34 (24-42) years. Prosocial personality traits were statistically significantly associated with scores on the DASS-21 subscale measuring depression: personal distress (PD) (r = 0.32; P = 0.01) and self-reported altruism (r = 0.30; P = 0.02). Only the subscale measuring the specific personality trait of personal distress (PD) was statistically significantly correlated with the scores of the other two DASS-21 subscales, anxiety and stress (anxiety: r = 0.54; P < 0.001; stress: 0.46; P < 0.001). Helpfulness was negatively related to anxiety (b = -0.29; P = 0.03).

**Conclusions:**

This research partially confirmed the hypothesis that stronger prosocial personality traits are associated with a lower prevalence of symptoms of depression, anxiety and stress in the MST of employees in a psychiatric clinic.

**Disclosure of Interest:**

None Declared

